# Unveiling exosomes: Cutting‐edge isolation techniques and their therapeutic potential

**DOI:** 10.1111/jcmm.70139

**Published:** 2024-10-21

**Authors:** Farnaz Sani, Faezeh Shafiei, Farshad Dehghani, Yasaman Mohammadi, Mohammadhossein Khorraminejad‐Shirazi, Abbas Fazel Anvari‐Yazdi, Zahra Moayedfard, Negar Azarpira, Mahsa Sani

**Affiliations:** ^1^ Shiraz Institute for Stem Cell & Regenerative Medicine Shiraz University of Medical Sciences Shiraz Iran; ^2^ Pharmaceutical Sciences Research Center Shiraz University of Medical Science Shiraz Iran; ^3^ Department of Pathology, School of Medicine Shiraz University of Medical Sciences Shiraz Iran; ^4^ Student Research Committee Shiraz University of Medical Sciences Shiraz Iran; ^5^ Department of Pathology, School of Medicine Jahrom University of Medical Sciences Jahrom Iran; ^6^ Division of Biomedical Engineering University of Saskatchewan Saskatoon Canada; ^7^ Department of Tissue Engineering and Applied Cell Sciences, School of Advanced Medical Sciences and Technologies Shiraz University of Medical Sciences Shiraz Iran; ^8^ Transplant Research Center Shiraz University of Medical Sciences Shiraz Iran

**Keywords:** cancer therapy, characterization, exosome, immune modulation, isolation

## Abstract

Exosomes are one type of nanosized membrane vesicles with an endocytic origin. They are secreted by almost all cell types and play diverse functional roles. It is essential for research purposes to differentiate exosomes from microvesicles and isolate them from other components in a fluid sample or cell culture medium. Exosomes are important mediators in cell–cell communication. They deliver their cargos, such as mRNA transcripts, microRNA, lipids, cytosolic and membrane proteins and enzymes, to target cells with or without physical connections between cells. They are highly heterogeneous in size, and their biological functions can vary depending on the cell type, their ability to interact with recipient cells and transport their contents, and the environment in which they are produced. This review summarized the recent progress in exosome isolation and characterization techniques. Moreover, we review the therapeutic approaches, biological functions of exosomes in disease progression, tumour metastasis regulation, immune regulation and some ongoing clinical trials.

## 1 INTRODUCTION

Medical technologies have advanced significantly in the 21st century, yet early diagnosis and full recovery from many diseases, including malignant tumours, remain major challenges. Over the past few decades, liquid biopsies have emerged as powerful non‐invasive diagnostic tools with broad potential for detecting various diseases early. Liquid biopsies involve the analysis of biological fluids from patients, such as blood, urine or cerebrospinal fluid (CSF), to gather disease‐related information. Key components include circulating tumour cells, exosomes and other extracellular vesicles (EVs).^1^ Among these, exosomes stand out due to their wealth of physiological and pathological information, making them useful across a wide range of diseases, from cancer to neurodegenerative and cardiovascular disorders. Exosomes are small vesicles, generally 30–120 nm in size originating from endocytic pathways. Initially discovered in reticulocytes as a mechanism to discard transferrin receptors.^2^, ^3^, ^4^ They are now known to be present in various biological fluids, including plasma, CSF, urine, saliva, semen and breast milk. Exosomes are secreted by all cell types and perform various functions, such as cell‐to‐cell communication and immune regulation.^5^ For effective research and therapeutic use, distinguishing exosomes from other EVs like microvesicles, and isolating them from complex biological samples, is crucial.

Exosomes have become promising tools for monitoring not only cancer but also other diseases, including cardiovascular conditions, neurological disorders and inflammatory diseases. Despite their potential, research on exosomes is still in its early stages. One significant challenge is their inherent heterogeneity, which makes it difficult to develop standardized isolation methods capable of targeting specific exosome subpopulations. To maximize their diagnostic and therapeutic applications, future exosome isolation strategies must focus on improving purity, throughput, operational efficiency and reproducibility.

This review covers recent advancements in exosome isolation techniques, characterization and biological functions. It also explores their potential applications in various therapeutic areas and discusses future directions for using exosomes in diagnosing and treating a wide range of diseases.

## 2 EXOSOME ISOLATION

**FIGURE 1:**
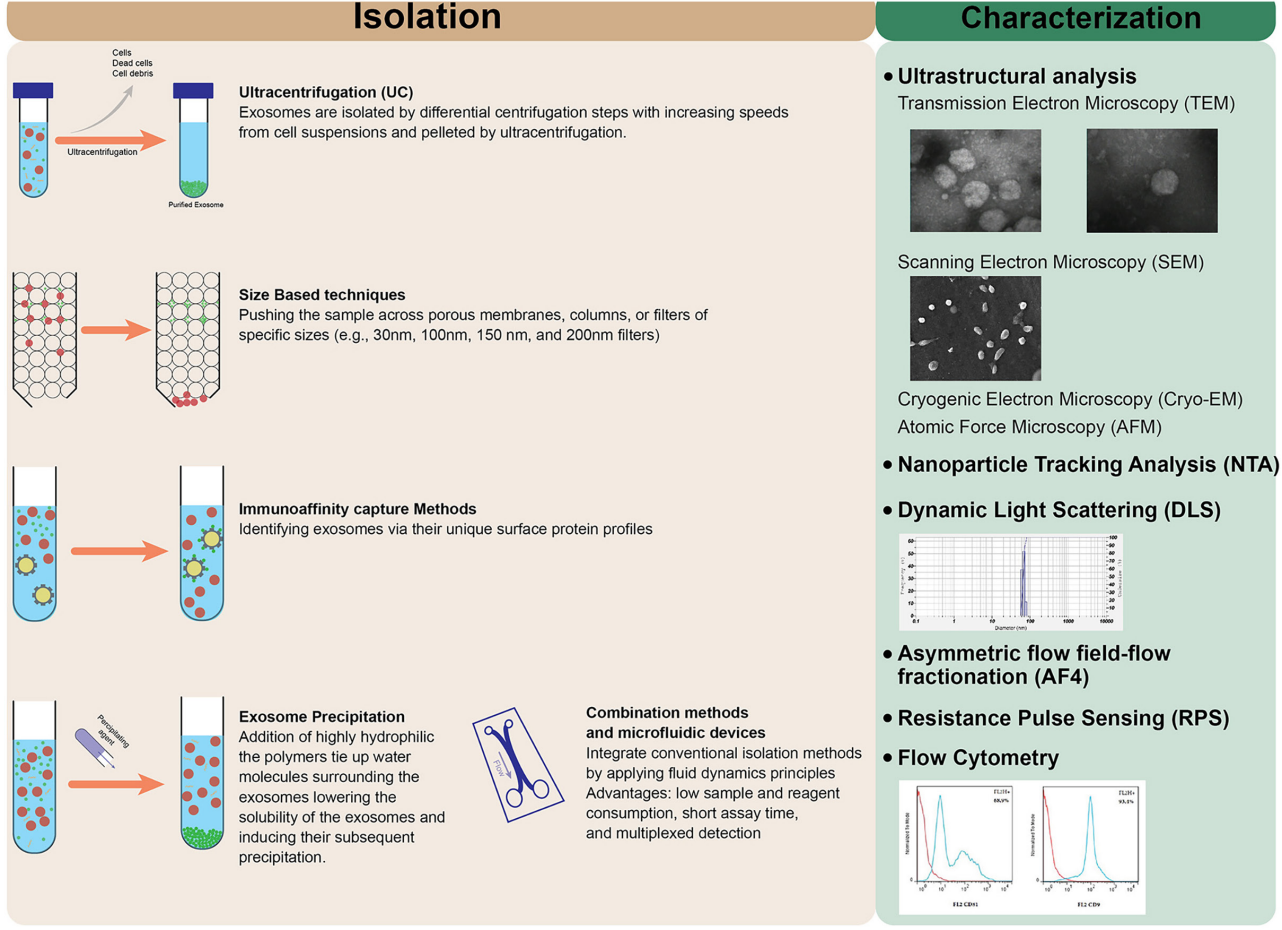
Schematic overview of common Methods for identifying, characterizing and analysing the functional properties of exosomes.

Several isolation techniques have been employed for whole exosome extraction, exosome RNA extraction or exosome protein profiling. Afterward, the purity and yield of exosomes can be measured using methods such as electron microscopy, flow cytometry, western blotting and liquid chromatography‐mass spectrometry (LC–MS).^6^ Some techniques have been routinely used to isolate and characterize exosomes (Figure 1).

The various isolation techniques have their use cases, and often, for a specific purpose, one isolation technique might be advantageous to others in terms of efficiency, yield, concentration and cost. We summarized some of the advantages and disadvantages of these methods in Table 1.

**TABLE 1:** Exosome isolation techniques.

Technique	Time [min]	Sample Types	Advantages	Disadvantages	Ref.
Ultracentrifugation: Differential UC Density Gradient UC (sucrose gradient, iodixanol gradient)	140–600	CCM, Urine, Plasma	Good for clinical applications and proteomics studies. Iodixanol gradient UC to separate exosomes from retroviruses	Impurities (proteins, RNA) Expensive and Complex Risk of damage to exosome integrity Long run time, especially gradient UC	8, 9, 10, 11
Size‐Based Techniques: UF, SF, SEC, FFFF, HFD SEC com. kits: qEV separation columns, EVSecond purification columns, Exo‐spin exosome purification columns	15–130	CCM, Urine	UF: fast, low‐cost, portable, simple SEC: high purity, low volume requirement, preserves exosome integrity, low‐cost com. kits available	UF: low purity, mechanical damage to exosome integrity, clogging of filter pores SEC: expensive and dedicated equipment, complex procedure	8, 9, 10, 11, 12, 13
Exosome Precipitation: ExoQuick (com.) TEI (com.) PEG	30–120	CCM, Plasma	High yield, high throughput, simple, portable, commercially available	Low purity (contamination)	8, 9, 10, 11, 13
Immunoaffinity capture: MagCapture ELISA	240	CCM	Excellent for isolation of tissue‐specific exosomes, very high purity	Expensive, Low yield, Long run‐time, requires preparation and preprocessing	10
Microfluidic devices: ExoChip Size exclusion chip (silicon‐nanowired) Viscoelastic Isolation Acoustic Isolation Electric ion separation Nano‐DLD nanoparticle sorting using pillar array chips	30–1200	CCM, Serum	Combination method based on size, density and immunoaffinity, Portable, fast (Acoustic isolation)	Non‐standard, low throughput, long run‐time (Silicon‐nanowired)	5, 7, 14, 15, 16, 17, 18, 19, 20

Abbreviations: CCM, cell culture medium; com, Commercial; FFFF, flow field‐flow fractionation; HFD, hydrostatic filtration dialysis; PEG, polyethylene glycol, SEC, size‐exclusion chromatography; SF, sequential filtration; TEI, total exosome isolation; UC, ultracentrifugation; UF, ultrafiltration.

### 2.1 Ultracentrifugation (UC)

Traditionally, UC was the first and default method of exosome extraction.^7^ In this method, cell culture medium (CCM) would be centrifuged sequentially at increasing spin speeds to remove cells and large and small particles. In this stage, spin speeds of 300 g up to 10,000 g would be utilized. In the next step, UC at spins of 70,000 g up to 200,000 g would be employed to isolate and further purify nanoparticles of 30–150 nm size, resulting in the highest concentration of exosomes with this method. To maximize concentration and minimize shearing forces of UC, various steps in this process have been replaced by other methods such as gradient‐based techniques, filtration, immune‐capture and chromatography‐based methods.^7^

**FIGURE 2:**
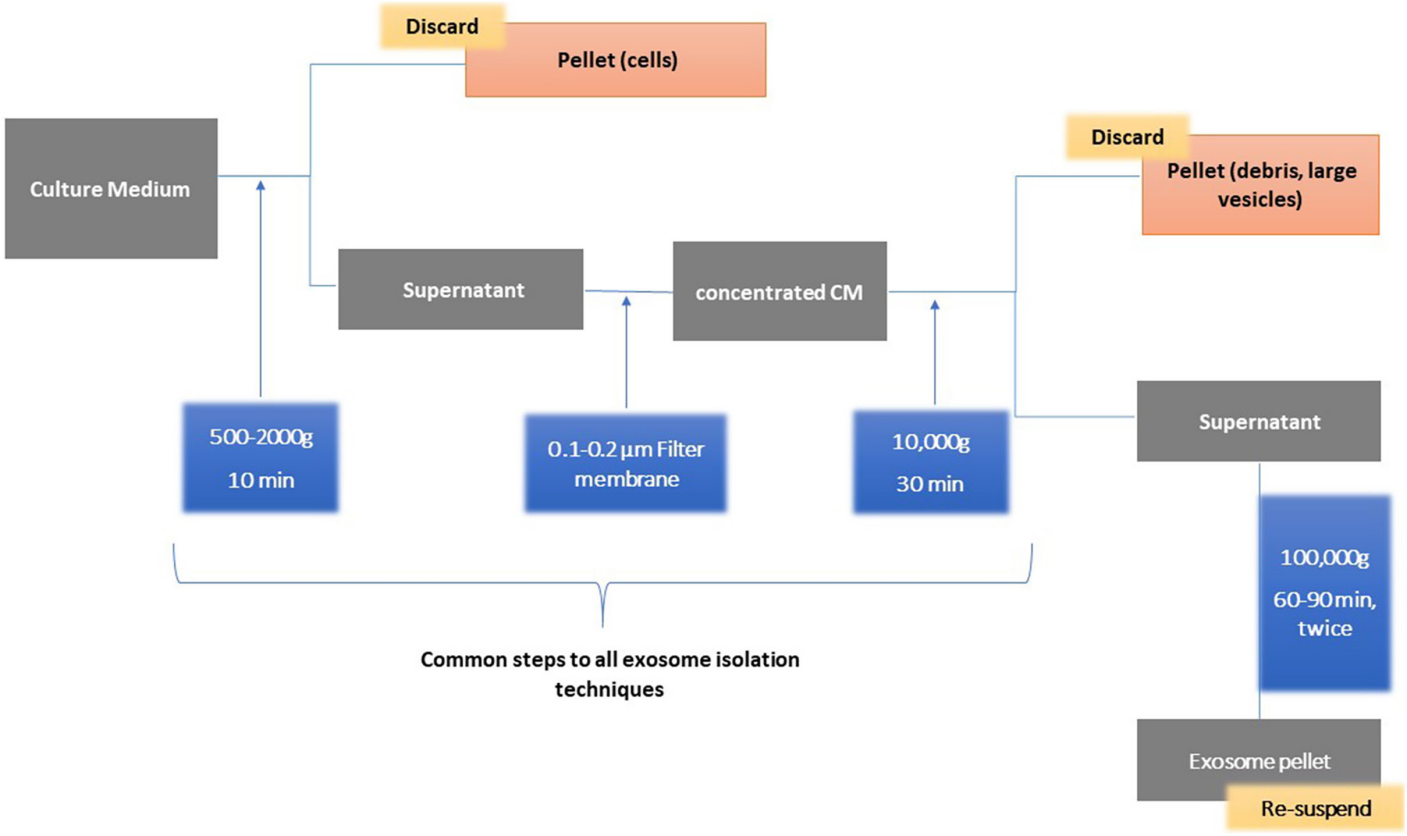
Differential UC. This technique commonly used in the field of biochemistry and molecular biology to separate and isolate different components of a biological sample based on their size, shape and density using high‐speed centrifugation.

The core principle here is the separation of CCM particles based on size, density and shape. In its simplest and oldest form, differential UC will employ ultraspins to separate particles in the medium. However, this method carries certain drawbacks despite its simplicity, making it undesirable in either clinical or research settings as a standalone exosome isolation method. UC can cause mechanical damage and rupture of exosomes due to the high physical pressure applied during the process. This can lead to contamination of the exosomal content with non‐exosomal macromolecules, reducing the yield of intact exosomes and complicating their characterization. Secondly, it is a time‐consuming method (several hours). Finally, it requires large starting volumes of samples (100 mL).^4^ The basic technical outline of this method is shown in this diagram (Figure 2):

Grad gradients have been utilized to separate particles in U. C. further to increase efficiency and yield. The sample will be passed through a prepared gradient (e.g. sucrose) in density gradient UC while subjected to specific centrifugal forces. This method, commonly employed in research, will greatly increase the efficiency and yield of exosomes while incurring additional time commitment.^8^, ^9^ UC has been regarded as the most reliable method for isolating small EVs. However, the unique complexity, composition and physical characteristics of each biofluid pose significant challenges to consistently achieving pure and reproducible EVs preparations. Additionally, factors such as rotor type, g‐force and centrifugation duration play a critical role in determining the yield of EVs in centrifugation‐based isolation methods. Thus, choosing the appropriate UC technique is essential for effective EVs isolation.

### 2.2 Size‐based techniques

Size‐based techniques include ultrafiltration, commercial exosome isolation kits, sequential filtration, size exclusion chromatography (SEC), flow field‐flow fractionation (FFFF) and hydrostatic filtration dialysis (HFD). The common theme among these, except FFFF, is pushing the sample across porous membranes, columns or filters of specific sizes (e.g. 30 nm, 100 nm, 150 nm and 200 nm filters) to exclude particles with molecular weights different from exosomes. These techniques don't usually require specialized and expensive equipment; commercial kits are available and have been employed in clinical settings. The downsides of this method include clogging of pores as well as mechanical damage to exosomes, leading to low purity.^8^, ^10^

**FIGURE 3:**
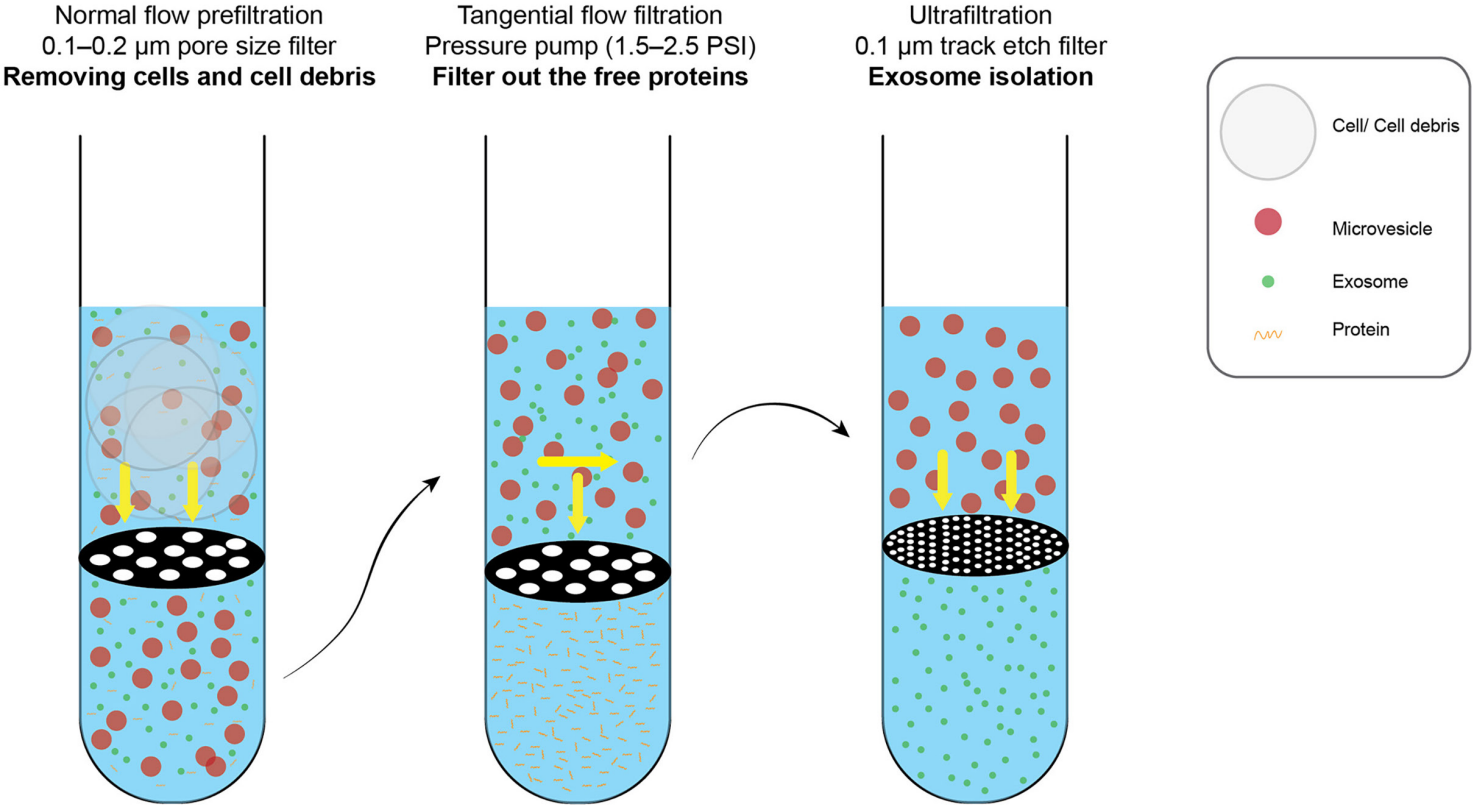
Sequential filtration. Sequential filtration begins by employing a filter with a relatively larger pore size, enabling the retention of larger particles while permitting smaller particles to pass through. The resulting filtrate from the initial filtration is then subjected to successive filtration stages utilizing filters featuring progressively smaller pore sizes. This iterative sequential filtration can be repeated several times to attain a more precise size‐based separation.

Sequential filtration uses multiple steps to filter out larger particles and cells in order to ultimately yield an exosome concentrate (Figure 3). In the case of a CCM sample, an exosome‐ or protein‐free medium (e.g. fetal bovine serum depleted of contaminants) is prepared, and cultured cells (e.g. breast cancer cells) are added to the medium and then incubated to allow for exosome release before proceeding with filtration steps. In the first stage, a 0.1–0.2 μm pore size filter is employed only to allow passage to exosomes and microvesicles (despite the larger particle size). In the following step, using a pressure pump (1.5–2.5 PSI), macromolecules are pushed out of the solution in the circulatory system of steps (discarding filtrates), ultimately retaining pure vesicles (exosomes and microvesicles). In the last step, a syringe pump (3.5 PSI transmembrane pressure) containing the sample is attached to a sterile 0.1 μm track etch filter (Millipore, Billerica, MA, USA) to retain microvesicles and filter out the exosomes.^11^

Flow field‐flow fractionation principally uses a perpendicular field force (flow‐based here) to nudge eluents out of their parabolic channel flow trajectory. This creates a difference in separation based on particle velocity and momentum, with smaller nanoparticles like exosomes travelling faster and eluting out of the channel sooner than bigger particles on a time scale. As particles precipitate along the lower channel wall, a counterflow pushes them back into the channel flow, creating a sequential particle separation based on their size and mass.^12^

Flow field‐flow fractionation has several notable limitations. The first is related to resolution; for instance, pure elution field‐flow fractionation (ElFFF) has largely been abandoned because it frequently produces poor resolution and signal issues, often caused by electrolysis byproducts and bubbles generated during experiments. Additionally, FFFF can struggle with complex mixtures, which can reduce its efficiency in separating components. To improve resolution, optimizing experimental conditions such as better controlling electrolysis and utilizing advanced instrumentation can significantly minimize signal distortions and enhance clarity. When dealing with complex samples, employing pre‐fractionation or using multiple FFFF stages with varied conditions can improve separation efficiency. Combining FFFF with complementary analytical techniques can further enhance the analysis of intricate mixtures. To address the limitations of conventional filtration, Chernyshev et al. developed asymmetric depth filtration (DF), a method that offers high yield and low contamination of small extracellular vesicles (sEVs). Conventional filtration typically includes surface filtration and DF. In surface filtration, large particles are retained on the surface, eventually forming a ‘cake’ of material, while DF media, with larger voids, allow particles to penetrate into the medium's pores. The asymmetric DF method operates on the principle that small particles can be eluted while sEVs are immobilized on the surface and within the depth of the porous media. This technique is particularly effective for isolating therapeutic sEVs from large volumes of growth medium used to culture EV‐secreting producer cells.^13^

### 2.3 Immunoaffinity capture methods

These techniques aim to identify exosomes via their unique surface protein profiles. A common method is to use a biotinylated antibody to capture exosomes in the sample, then utilize the avidin‐coated surface and avidin‐biotin interaction to isolate the antibody–antigen complex.

Exosomes have highly specific surface antigens that can be isolated with immune‐capture methods. In addition, specific exosomes of a certain tissue can be further distinguished by their native antigens, making this excellent for targeted studies. For a surface exosome protein to be used for immunoaffinity capture, the said protein should be exclusively or highly expressed on the exosome surface and not present in the extracellular fluid. Several transmembrane, receptor, heat shock and lysosome‐associated proteins have been profiled for exosome research. Among these, transmembrane proteins such as Rab5, CD81, CD63, CD9, CD82, annexin and Alix have been extensively exploited for selective exosome isolation.^14^, ^15^ Despite these advantages, this method is unsuitable for high‐yield throughput purposes.^8^

Several commercial kits are available in this category, including exosome‐human EpCAM isolation reagent (*Thermo Fisher Scientific* **)**, exosome isolation and analysis kit (Abcam), exosome‐human CD63 isolation reagent (Thermofisher), and exosome isolation kit CD81/CD63 (Miltenyi Biotec).^14^

Benecke et al. found that the EVs isolated using the immunomagnetic bead‐based technique known as ‘EXOBead’ exhibited greater purity than those obtained through size‐exclusion methods.^16^ However, several factors can influence the effectiveness of this isolation, including the high cost of antibodies, the extended time required for antibodies to bind to the EV surface antigens, and the conditions used for elution. Additionally, the choice of elution buffer can impact the structural integrity of the EVs. The quantity of isolated EVs may also be restricted by the availability of binding molecules, making it more challenging to retrieve intact EVs from the antibody beads. In a different approach, Yoshida et al. developed a TIM4‐affinity isolation technique that specifically targets phosphatidylserine (PS), which is a key component of the EV membrane. TIM4 interacts with PS through calcium‐dependent binding, enabling the elution of intact EVs from the TIM4 beads when using the chelating agent ethylenediaminetetraacetic acid. This TIM4‐affinity method addresses some of the limitations associated with traditional affinity isolation techniques and facilitates the high‐purity isolation of diverse EVs.^17^

### 2.4 Exosome precipitation

The precipitation technique involves the addition of polymers to a sample intended for isolation, forming a mesh structure with water molecules and sEVs during low‐speed centrifugation. This approach was initially developed for isolating viruses and has since been adapted for extracting due to their comparable sizes. Both natural (such as chitosan) and synthetic polymers (like polyethylene glycol, or PEG) can be utilized, with PEG being the most frequently used option because of its low toxicity and ability to be modified for water solubility.^18^

An enhanced PEG precipitation method known as ‘ExtraPEG’ was proposed by Rider et al., which facilitates the rapid and large‐scale enrichment of EVs. The EVs obtained through this method were found to be biologically intact and demonstrated superior performance compared to those purified with a commercial kit (ExoQuick). Natural polymers tend to be more biocompatible, nonimmunogenic, biodegradable and less toxic than synthetic options, making them more suitable for clinical applications. Kumar et al. showed that chitosan can effectively isolate EVs from diverse biological samples by forming a complex with the EVs that subsequently sediments during centrifugation. However, the higher cost of natural polymers poses a challenge.

In general, polymer precipitation offers several benefits, including simplicity, speed and ease of handling without requiring expensive equipment, making it a practical choice for large‐scale production. Nevertheless, while the yield of EVs obtained through PEG precipitation is significantly enhanced, this method also leads to increased contamination, resulting in lower purity. This can impact subsequent analyses of EVs, necessitating further purification via SEC. Currently available polymer precipitation kits include the ExoQuick kit from System Biosciences and the Total Exosome Isolation Kit from Thermo Fisher Scientific.^19^, ^20^, ^21^

### 2.5 Combination methods and microfluidic devices

Microfluidic devices are lab‐on‐chip technologies developed in the 1980s to analyse and manipulate fluids at a microscopic scale. They use the physical, electrical and immune affinity properties of macromolecules and particles to separate them.^22^ In a recent 2020 study, Han et al. utilized Morpho Menelaus (a species of butterfly) wings that generate micro vortices resembling herringbone properties that can effectively capture and isolate exosomes (>70% isolation efficiency) when coated with fluorescein isothiocyanate (FITC) labelled lipid nanoprobes (DSPE‐85 PEG‐FITC) and treated with 3‐Aminopropyltriethoxysilane (APTES). Menelaus wings can enhance the fluorescence intensity, which is advantageous for biological detection.^23^ Potential commercial kits have been developed using microfluidic devices. Kanwar et al. fabricated a polydimethylsiloxane (PDMS) microchip using standard soft lithography techniques and then treated it with surface functionalization chemicals including 3‐mercaptopropyltrimethoxysilane (Gelest), GMBS (Pierce), NeutrAvidin (Invitrogen) and biotinylated anti‐CD63 (Ancell). This ‘ExoChip’ was designed as a series of small circular chambers connected by straight channels. The slow flow in the chambers allows sufficient contact between exosomes and anti‐CD63 antibodies. One advantage of this device is the immediate quantification of exosomes using fluorescent microscopy on the ‘ExoChips’. Using these chips, they detected a 2.34‐fold increase in exosomes captured in patients with cancer compared to healthy individuals.^24^ Mogi et al. utilized an ion exchange membrane (Nafion, 274,704‐25ML, Sigma‐Aldrich) between two microchannels in a PDMS chip to separate charged particles and create a trans‐channel voltage. Nafion would only allow one‐way passage to cations, creating negative charge excess in the main channel, thus creating an ion‐depletion zone around the entry points where only non‐charged particles can accumulate (and cations pass through). As exosomes are negatively charged, they are deflected by this zone and separated from other non‐charged particles. The size of the ion‐depletion zone depends on various factors, such as inlet flow rate and cross‐membrane voltage. Perhaps the biggest advantage of this method is its extremely high exosome yield (98%), demonstrating minimal structural damage to exosomes.^25^ IBM scientists used microfluidic micropillar arrays in a deterministic lateral displacement (DLD) formation to separate particles between 20 and 110 nm in size based on their zigzag movement across the array, in contrast with the bumping movement of larger particles.^26^

Combining different methods has been very beneficial in maximizing purity and yield by eliminating the weakness of a sub‐step by employing an advantageous method. For example, SEC could be utilized as a further step towards purifying UC results. Immune capture methods are often performed after initial isolation via UC, UF or precipitation methods. It is worth mentioning that selecting an appropriate method also depends on the sample characteristics, as certain techniques are not suitable for all mediums.^6^

### 2.6 Characterization of exosomes

Several characterization methods have been developed for research and clinical purposes to analyse exosome quantification, visualization and purity. These methods include ultrastructural analysis, nanoparticle tracking analysis (NTA), dynamic light scattering (DLS), asymmetric flow field‐flow fractionation (AF4), resistance pulse sensing (RPS) and flow cytometry. The ultrastructural analysis includes transmission electron microscopy (TEM), scanning electron microscopy (SEM), cryogenic electron microscopy (Cryo‐EM) and atomic force microscopy (AFM) techniques. We review these current practical techniques and address their characteristics and experimental limitations here.

#### 2.6.1 Ultrastructural analysis

##### Transmission electron microscopy (TEM)

Transmission electron microscopy (TEM) is the optimum method to study exosome structure, morphology and size. The negative staining procedure is uncomplicated and rapid, lasting only two to 3 h and the resolution for TEM is about 1 nm.^27^ Exosomes are fixed in 2% paraformaldehyde, placed on Formvar carbon‐coated TEM grids, and incubated for 20 min. The grids are then washed with phosphate‐buffered saline (PBS), incubated with glutaraldehyde and washed with water. The vesicles are then stained with a uranyl acetate solution and air‐dried.^28^ The TEM technique is based on creating images as a beam of electrons passes through a specific sample, where a secondary electron is generated, and the electrons are collected and magnified using special lenses.

TEM is a valuable tool for detecting and characterizing individual sEVs, revealing critical information about sample purity and molecular composition. While conventional TEM can achieve a high resolution of 1 nm, it often damages biological samples through preparation methods that involve dehydration, freezing or adsorption. Additionally, the electron microscopy (EM) characterization of EVs necessitates costly equipment and specialized training to operate both the hardware and software effectively.

Cryo‐transmission electron microscopy (cryo‐TEM) addresses many of the limitations associated with traditional TEM. This technique allows for the visualization of surface morphology, making it particularly effective for distinguishing vesicles based on their surface characteristics. As a result, cryo‐TEM facilitates the differentiation of various vesicle subpopulations and aids in determining their size distributions.^29^, ^30^

##### Scanning electron microscopy (SEM)

Scanning electron microscopy (SEM) uses a fine‐point beam, which scans samples line by line, unlike using a broad beam in the TEM method. Therefore, SEM focuses on the surface of samples to provide a three‐dimensional image of exosomes rather than the two‐dimensional image generated by TEM. Exosomes are fixed with glutaraldehyde and dehydrated with an ascending sequence of ethanol. Exosomes are ready for SEM analysis after air‐drying samples at room temperature.^31^ Sharma et al. indicated that, unlike the cup‐shaped exosomes observed under TEM, round and bulging exosomes without a central depression were shown by SEM.^32^ SEM helps maintain the moisture levels in cells; however, it can alter the structure of fragile protrusions, leading to their collapse. In contrast, cryo‐electron tomography (cryo‐ET) prevents alterations in ultrastructure and the redistribution of cellular components.

##### Cryogenic electron microscopy (Cryo‐EM)

Cryogenic electron microscopy (Cryo‐EM) is a type of electron microscopy that allows samples to remain in their native aqueous forms while the cells are intact, with no ultrastructural changes or redistribution of elements. Suspended exosomes are deposited on a grid, which is then rapidly immersed in liquid ethane to allow vitrification of the sample. After vitrifying, samples are analysed under cryo‐EM or transferred to liquid nitrogen for storage.^33^ Yuana et al. reported that exosomes have a clear bilayer structure and are sometimes surrounded by smaller vesicles under the cryo‐EM method.^34^ Furthermore, specimens are safeguarded from damage due to electron beam radiation by utilizing extremely low temperatures. In low‐dosing techniques, inelastic scattering can create significant background noise in images, but this can be mitigated by enhancing the signal‐to‐noise ratio, enabling computer‐assisted higher resolution for single‐particle analysis. The conventional approach for cell preparation routine electron microscopy (EM), which includes fixation, dehydration, embedding and sectioning is employed to minimize the drying effects on sEVs. Routine EM with plastic embedding can help reduce or eliminate artefacts related to denaturation, such as changes in volume and shape. Glutaraldehyde is often used for chemical fixation to facilitate crosslinking through covalent bonds between amino groups, while osmium tetroxide is typically employed to fix lipids and enhance image contrast. Additionally, cryo‐electron tomography (cryo‐ET) can reveal the ultrastructural details of EVs at high resolution; however, its application is generally limited to areas of specimens less than 500 nm thick. The inelastic mean‐free path of 300 keV electrons in biological samples embedded in vitreous ice exceeds 300 nm.^28^, ^35^, ^36^

##### Atomic force microscopy (AFM)

Atomic Force Microscopy (AFM) detects and records interactions between a probing tip and the sample surface. The substantial feature of this technique is its ability to measure samples in native conditions with minimal sample preparation and without any destructive mode of operation.^37^, ^38^ This method has a high resolution of about 1 nm and is suitable for exosome topography studies.^27^ The exosome suspension is placed on a mica substrate and then air‐dried at room temperature. Samples are then washed with ultrapure water and dried with nitrogen gas. Finally, samples are viewed using AFM with silicon probes and analysed with AFM software.

The main techniques for nanoscale imaging with atomic force microscopy (AFM) are contact mode and tapping mode. In contact mode, a sharp tip directly contacts the sample surface, gathering topographical information while maintaining a consistent force in the nano newton range. Tapping mode, on the other hand, oscillates the tip at its resonant frequency, resulting in brief contact that minimizes interaction forces, making it ideal for fragile surfaces and enhancing imaging repeatability and resolution. This makes the tapping mode preferable for imaging soft biological samples.^39^, ^40^, ^41^ Despite careful data processing, significant errors can occur in surface topography measurements due to convolution effects from the probe's finite size. Research has focused on reconstructing true surfaces from distorted images. Reiss et al. studied the impact of tip size, while Keller introduced Legendre transforms to deconvolve the tip's influence. Villarrubia proposed a mathematical morphology approach to address convolution challenges.^42^, ^43^

This method models the tip and sample assets of peaks, allowing flexibility based on the number of peaks for resolution. However, mathematical morphology techniques face limitations, such as lengthy reconstruction times and the need for detailed tip and sample information. They often assume symmetrical surface features, using geometric approximations to estimate dimensions, which complicates accurate surface reconstruction. To overcome these challenges, Canet‐Ferrer et al. developed a simple algorithm for reconstructing AFM images that simulates convolution effects and applies them to various nanostructures.^44^, ^45^

## 3 NANOPARTICLE TRACKING ANALYSIS (NTA)

Nanoparticle Tracking Analysis is a high‐resolution technique capable of measuring exosome concentration and size distribution within the 10 nm to 2 μm range. It tracks the movement of exosomes and measures their velocity by monitoring the Brownian motion of nanoparticles in a liquid suspension on a particle‐by‐particle basis through image analysis, allowing movement to be correlated with particle size.^46^, ^47^ During NTA measurements, sEVs are visualized by the light they scatter when illuminated by a laser beam. This scattered light is focused through a microscope onto a camera that records the particles' movement, and the NTA software analyses this motion to calculate the diffusion coefficient for each particle using the Stokes‐Einstein equation.^48^ A typical NTA setup includes a laser module, a microscope connected to a sensitive charge‐coupled device or complementary metal‐oxide semiconductor camera, a hydraulic pump and a measurement chamber.^49^, ^50^

NTA has several advantages for detecting various EVs. It can accurately measure particles as small as 10 nm–2 μm range in diameter, and sample collection occurs in the liquid phase, reducing variations in the EVs being analysed. Sample preparation is simple, with measurements taking only a few minutes, and the sample can be recovered in its natural state after analysis, enhancing the technique's attractiveness.

Additionally, the NTA system can detect fluorescence, enabling the identification of antigens on sEVs through fluorescently labelled antibodies. This feature allows for the examination of antigenic composition and size distribution in smaller EVs, providing insights not achievable with other methods and offering potential for monitoring phenotypic changes in EVs related to disease.^51^

In contrast to NTA, DLS tends to favour larger particles, which can obscure smaller ones, whereas NTA provides more accurate measurements by focusing on individual particles.^52^ However, NTA has limitations, such as the challenge of determining the appropriate dilution of the final sample. Finding the right dilution is essential to ensure that the NTA camera can capture all EVs without larger particles masking the smaller ones. Similar to other methods relying on Brownian motion, larger vesicles can obscure the results of smaller ones, leading to unreliable data.^53^ Additionally, while NTA can detect fluorescence signals, its effective application for sEV phenotyping is limited by the requirement for sufficiently strong fluorescence signals to be detected by current NTA systems.

### 3.1 Dynamic light scattering (DLS)

Dynamic light scattering, also known as photon correlation spectroscopy, is an alternative method for measuring the size of exosomes. The procedure of DLS is that a monochromatic coherent laser beam passes through a suspension of particles.^54^ Time‐dependent fluctuations in scattering intensity caused by constructive and destructive interference resulting from the Brownian movements of the particles within a sample are then perceived. This method is simple to use, but it does not visualize the particles. The advantage of using this method is its ability to measure particles ranging in size from 1 nm to 6 μm.^55^

In comparison to single‐particle imaging techniques, DLS can gather data on numerous particles quickly, allowing for the analysis of large sample batches. Additionally, it requires only a small amount of sample, which can be reused, and the sample preparation process is straightforward and non‐invasive. However, DLS yields an average measurement for relatively uniform particles, making it less suitable for assessing heterogeneous solutions of EVs. While DLS can measure sEV diameters ranging from 1 nm to 6 μm, it does not provide biochemical information or details about the originating cells.^49^ Importantly, DLS is significantly less accurate when dealing with heterogeneous mixtures of EVs, leading to less precise results.^56^, ^57^ The DLS signal relies on the size and concentration of macromolecules, so optimizing the concentration range of EVs may be essential for obtaining reliable measurements.

### 3.2 Asymmetric flow field‐flow fractionation (AF4)

This method separates exosomes based on their density and hydrodynamic properties. Exosomes flow through a forward laminar channel and are sorted into separate populations based on their Brownian motion. Smaller particles have higher diffusion rates and tend to move faster; however, larger particles have lower diffusion rates and tend to move more slowly. In contrast to NTA, which in prior studies resolved only a single, broad peak from 50 to 150 nm, AF4 was able to distinguish two separate exosome subpopulations: large exosome vesicles with sizes ranging from 90 to 150 nm and small exosome vesicles with sizes around 30 nm. Hence, AF4 better addresses the size heterogeneity of exosome samples and can be considered an advanced analytical technique for exosome subset characterization.^58^, ^59^, ^60^

### 3.3 Resistance pulse sensing (RPS)

The Resistance Pulse Sensing (RPS) technique measures the size of exosomes based on their electrical resistance when they pass through a small orifice. This technique detects vesicles with diameters of 50–1000 nm. The important feature of RPS is the single‐particle characterization and concentration measurement of exosomes. The drawback of this technique is that its measurements are susceptible to system stability and sensitivity issues, where the pore can become blocked by particles and where particles are too small to be detected against the background noise of the system subsequently. The conducted studies have demonstrated that system sensitivity and stability can be improved by optimizing system parameters, such as system noise, sensitivity cut‐off limits and accuracy.^61^, ^62^

RPS was introduced in 1976 for the detection and characterization of viruses.^63^ The technique known as tunable resistive pulse sensing (TRPS) operates on the Coulter principle, enabling the detection, measurement and analysis of particles ranging from tens of nanometres to micrometres. With advancements in this method, TRPS is now employed for characterizing EVs.^64^

In TRPS, a voltage is applied across a membrane with pores, causing the sample to move to one side. As individual particles pass through these pores due to the pressure differential and voltage, the current flowing through the holes momentarily decreases. This occurs because the particles possess higher electrical resistance than the surrounding electrolyte, allowing for the determination of EV concentration and size. The concentration is derived from the frequency of particle passage through the membrane, while the size is inferred from the reduction in current. The membranes used in TRPS are flexible, and their pore sizes can be adjusted by stretching, enhancing the technique's sensitivity and accuracy for different samples.^65^

TRPS offers the advantage of rapid sampling compared to optical sensing methods like NTA. However, it may encounter issues such as pore clogging and decreased sensitivity when the transfer rate through the holes increases or when sampling frequency and bandwidth decrease during sEV quantification.^66^

### 3.4 Flow cytometry

The flow cytometry technique is a molecular approach utilized to characterize exosomes' surface proteins, and the size and structure of exosomes are measured by this technique.^67^ The flow cytometry technique is well‐adapted to the reproducible analysis of clinical samples, and different physical and chemical characteristics of cells and particles in suspension are analysed, and the size and structure of exosomes are measured.^68^ Conventional flow cytometers measure particles greater than 300 nm, while they cannot detect smaller particles based on forward scattered light (FSC). Hence, these instruments do not allow the direct detection of exosomes.

Flow cytometry is a highly effective tool for analysing individual particles; however, its instruments and techniques were primarily designed for cell analysis, making them less suitable for examining small, faint EVs. Additionally, the detection thresholds of commercially available flow cytometers can vary widely. As a result, flow cytometry has not achieved the same level of acceptance for EV analysis as it has for cell studies. There are also notable discrepancies in sample preparation and measurement protocols, with little consensus on standardization, calibration and experimental design principles, as well as inconsistencies in data reporting and storage. The new generation of flow cytometers utilizes multiple angles for FSC detection, which improves particle resolution. The salient features of this technique are rapid measurement of suspended exosomes, detection of EVs smaller than 300 nm, and quantification and/or classification of exosomes according to the antigen expression level.^69^

## 4 FUNCTIONS AND CLINICAL APPLICATIONS OF EXOSOMES

Exosomes participate as an important mediator in cell–cell communication and deliver their cargos, such as mRNA transcripts, microRNA, lipids, cytosolic and membrane proteins, and enzymes, to target cells with or without physical connections between cells.^70^, ^71^, ^72^, ^73^ Exosomes are highly heterogeneous in size, and their biological functions can vary depending on the cell type, their ability to interact with recipient cells and transport their contents, and the environment in which they are produced.^58^, ^70^, ^74^, ^75^, ^76^, ^77^ Among the markers of exosomes are proteins such as CD9, CD63 and CD81 from the tetraspanin family, along with heat shock proteins (Hsp), actin, flotillins, the endosomal sorting complex required for transport proteins (Alix and TSG101), and integrins, resulting in the specificity of exosomes.^6^, ^78^

It is essential to understand that exosomes reflect the traits of their source cells; those produced in disease contexts tend to exacerbate pathological conditions, while those from progenitor cells possess healing and restorative functions. Studies indicate that exosomes are vital to a wide range of physiological processes and are significantly involved in the mechanisms underlying various diseases, such as neurodegeneration, cardiovascular disorders, cancer, autoimmune diseases and infections.^79^, ^80^, ^81^, ^82^, ^83^, ^84^, ^85^ Furthermore, exosomes play a crucial role in tissue repair, stem cell maintenance, tumour progression through angiogenic stimulation and metastatic tumour cell migration, and in cellular biological processes such as immunosuppressive differentiation of regulatory T lymphocytes or myeloid cells, inflammation, apoptosis, coagulation, antigen presentation and intercellular signalling.^71^, ^73^, ^86^, ^87^, ^88^, ^89^, ^90^, ^91^ The distinctive attributes of exosomes facilitate inflammation reduction, blood–brain barrier penetration, multiple intravenous (IV) dosing without adverse effects, and enhancement of neural and motor function.^70^ Given these properties, exosomes have become the subject of extensive scientific investigation. They are particularly seen as favourable biomarkers for diagnosis and predicting various diseases, potentially leading to the advancement of minimally invasive diagnostic techniques and next‐generation therapeutic interventions.^70^, ^92^ In this regard, analysis of data from ClinicalTrials.gov (https://clinicaltrials.gov/) reveals that exosomes find clinically prominent applications as biomarkers, exosome therapy agents, drug delivery systems and in the development of cancer vaccines.^93^

Mesenchymal stem cell (MSC)‐derived exosomes have been shown to possess anti‐inflammatory properties by inhibiting the expression of pro‐inflammatory cytokines and facilitating tissue regeneration through enhanced extracellular matrix remodelling.^70^, ^94^, ^95^, ^96^ Additionally, exosomes released from induced pluripotent stem cells, embryonic stem cells and cardiac progenitor cells exhibit therapeutic effects similar to those of MSC‐derived exosomes.^70^, ^97^, ^98^ Moreover, MSC‐EVs have been shown to replicate their parent cells' immunomodulatory and cytoprotective properties.^70^, ^99^ Interestingly, studies have revealed that the diverse therapeutic effects exhibited by exosomes from various sources highlight their promising role in regenerative medicine and as potential therapeutic agents for inflammatory and degenerative conditions.^70^ For example, bovine milk‐derived exosomes can attenuate arthritis,^100^ or exosomes derived from bone marrow MSCs demonstrate a protective role against diverse disease conditions, such as hypoxia‐induced pulmonary hypertension,^101^ myocardial ischemia/reperfusion injury,^102^ and brain injury.^103^ Moreover, EVs derived from human umbilical cord MSCs exhibit beneficial effects in mitigating acute renal injury and liver fibrosis,^104^, ^105^ DC‐derived exosomes exposed to cancer antigens induce a cancer‐specific T cell response,^106^ and exosomes from B16BL6 murine melanoma cells containing melanoma antigens trigger B16BL6‐specific T‐cell responses and inhibit tumour growth.^107^, ^108^

In addition, Exosome‐mediated cargo transfer has implications for the progression of various diseases. Therefore, developing exosome‐based therapies and diagnostic tools relies on thoroughly understanding exosome functions in disease pathways.^6^

Although the primary focus of ongoing exosome‐based clinical trials is on identifying diagnostic or prognostic biomarkers, an increasingly significant number of trials are also investigating exosomes as potential therapeutic agents in various diseases, including neurodegeneration, cardiovascular diseases, cancer, immunomodulation and infectious diseases.^6^ The primary sources for therapeutic exosomes are MSCs, dendritic cells (DCs) and even autologous tumour cells, whether in their unmodified or engineered form.^93^ In the beginning, we describe instances of exosome functionality in their native condition and then elaborate on the role of exosomes in disease pathways, along with the clinical utility of exosomes in diagnostics and therapeutics.

### 4.1 Exosomal membrane proteins in the natural pathway

Exosomes play pivotal roles in immune system regulation^109^ and have the capacity to influence immune responses by presenting antigens on their surfaces. For instance, exosomal membranes can merge with MHC‐antigen complexes, initiating and advancing inflammation through antigen‐specific T‐cell responses.^7^ The exosome surface receptors CD86 and lymphocyte function‐associated antigen 1 (LFA‐1) activate inflammatory pathways that stimulate immune cells.^110^ Moreover, exosome surface proteins facilitate immune suppression; exosomes derived from cancer cells expressing programmed death‐ligand 1 (PDL1), an inhibitory checkpoint molecule, can hinder cytotoxic T‐cell function^111^ and support the evasion of cancer cells from the immune system. Beyond surface proteins, exosomes are also known to carry protein, DNA and RNA cargo capable of triggering immune responses and other physiological functions.^112^

### 4.2 EV‐mediated cargo transfers from donor to recipient cells

Through the transfer of proteins, nucleic acids and lipids from donor to recipient cells, exosomes establish a newly recognized pathway for intercellular communication.^85^, ^113^ Particularly, the transport of exosomal cargo, notably nucleic acids, plays a key role in regulating the behaviour of the receiving cell.^72^, ^114^ For instance, miRNA‐containing exosomes derived from MSCs aid in mending injured myocardium, presenting a promising MSC alternative.^115^ Interestingly, MSC‐derived exosomes exhibit greater efficacy in preventing hypertrophy or damage compared to MSCs themselves, attributed to differences in miRNA content between exosomes and parental cells.^116^, ^117^

Exosomal delivery of soluble cytokines, growth factors and hormones emerges as a crucial mechanism for intercellular communication, enabling long‐range signalling and the modulation of systemic immune reactions.^118^, ^119^, ^120^ Moreover, the lipid cargo of exosomes displays diverse functions, influencing the metabolism and immune response of the recipient cells.^121^, ^122^

In cancer progression, exosomes derived from mesenchymal cells initiate metastasis through the epithelial‐mesenchymal transition, transforming adhesive epithelial cells into migratory, invasive mesenchymal cells.^123^ These mesenchymal‐derived exosomes carry distinct cargo compared to epithelial cells and actively contribute to tumour growth by promoting immune suppression by delivering proteins, lipids and nucleic acids.^124^ Notably, fatty acid cargo from tumour‐derived exosomes can suppress the immune responses of recipient DCs.^121^

### 4.3 Exosome as a therapeutic agent

**FIGURE 4:**
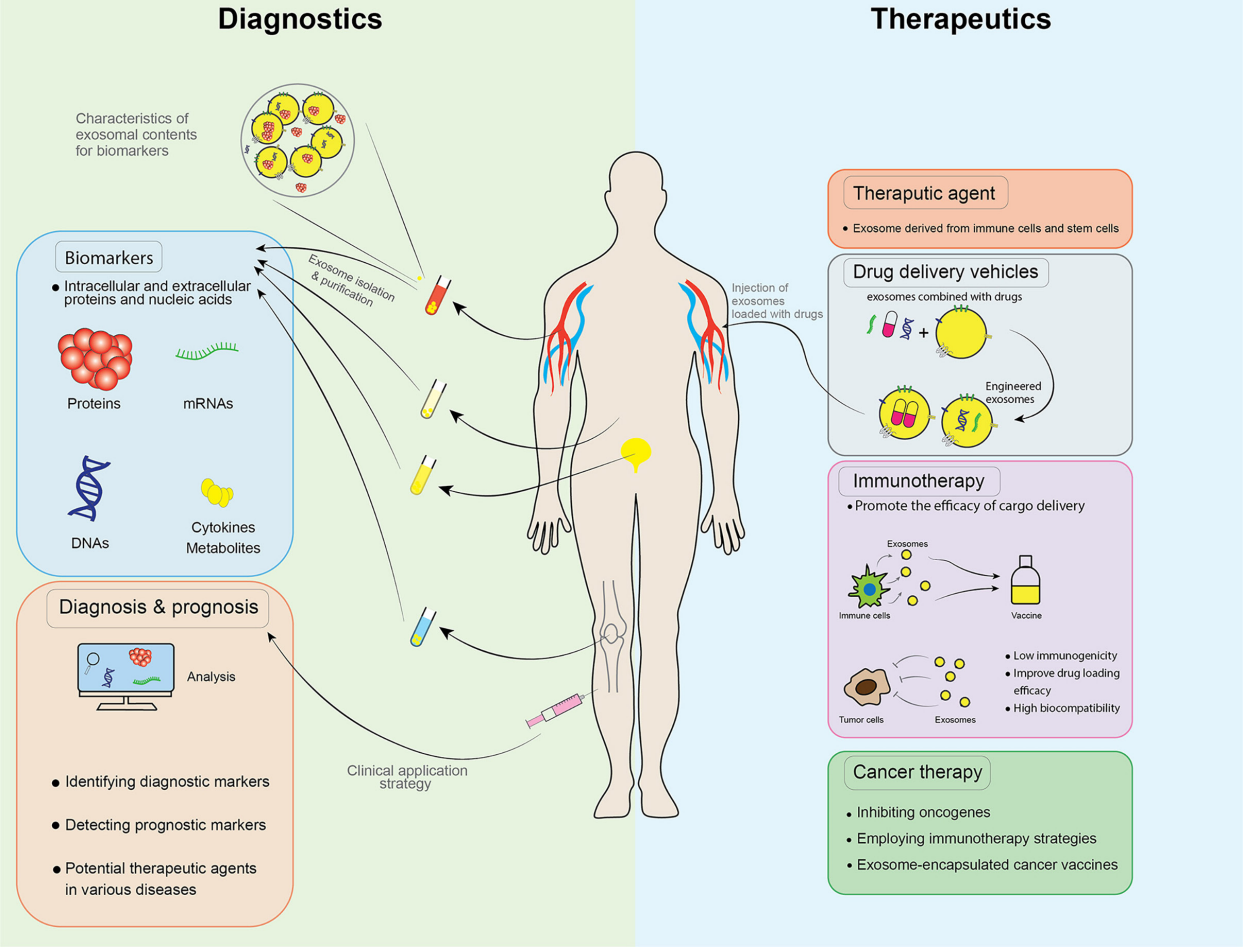
Clinical applications of exosomes. Exosomes can be extracted from bodily fluids, lymph, bile, blood, urine and etc. Analysing the molecular contents of exosomes can provide unique opportunities in their identifications. Exosomes can be used as biomarkers in disease diagnosis and surveillance. Clinical treatment mostly involves these strategies: First, cargo, including drugs, nucleic acids and proteins, can be encapsulated in exosomes and targeted to the mentioned sites. Also, immunotherapy can be used in cancer and other disease therapies.

Exosomes derived from immune cells and stem cells have shown potential for promoting immune responses and tissue regeneration, respectively.^125^, ^126^, ^127^ Clinical trials have utilized dendritic cell‐derived exosomes containing antigen presentation molecules and costimulatory molecules as immunotherapy agents for lung cancer patients, enhancing the anti‐tumour activity of natural killer cells.^128^, ^129^ Studies have also revealed that exosomes from disease‐ or infection‐related cells can stimulate immune responses.^128^, ^130^ For instance, exosomes from lung carcinoma cells altered after respiratory syncytial virus infection induced an innate immune response through enhanced cytokine and chemokine release. These findings highlight the therapeutic potential of exosomes in novel approaches against various diseases and injuries (Figure 4).

#### 4.3.1 Cancer therapy

Various exosome‐based approaches are being explored as potential cancer therapies, focusing on inhibiting oncogenes and employing immunotherapy strategies For instance, a phase I trial (NCT03608631) was conducted at M.D. Anderson Cancer Center (Texas, USA) investigates using MSC‐derived exosomes to treat stage IV pancreatic cancer patients with the KrasG12D mutation. In this trial, patients are administered KrasG12D‐specific siRNA‐loaded exosomes to target the oncogenic KRAS gene and reduce its expression in pancreatic tumours.^131^

Immunotherapy has also been tested in patients with unresectable non‐small cell lung cancer in a trial (NCT01159288) involving dendritic cell‐derived exosomes loaded with tumour antigens.^132^ While specific T‐cell responses against cancer cells expressing the antigen of interest were not observed, some patients experienced a significant increase in NK cell activation. However, the primary endpoint of achieving 50% nonprogressors was not met, and the trial was terminated.

Tumour cells represent a promising source of exosomes in cancer therapy due to their tropism and ability to induce a specific inflammatory response. Using the patient's tumour cells as the manufacturing cell line offers the advantage of preventing neutralization by innate immunity. In a phase I trial (NCT01550523), autologous glioma cells pretreated with an anti‐sense molecule targeting the tumour's tyrosine kinase cell surface receptors (ILF1R) were employed to inhibit tumorigenesis. As of now, no results from this trial have been disclosed.^133^ These exosome‐based strategies hold potential in cancer therapy, but further research and clinical trials are essential to fully understanding their efficacy and safety in treating various cancer types.

#### 4.3.2 Exosome‐encapsulated cancer vaccines

Exosomes derived from tumour cells have shown promise as therapeutics to elicit robust anti‐tumour immune responses. Additionally, exosome‐based vaccines have demonstrated encouraging outcomes against various infectious diseases. These exosomes, originating from innate immune cells and tumour cells, hold potential as cancer vaccines due to their active molecules, such as MHC and costimulatory molecules, that facilitate immune responses against tumours.^134^ Profiling exosomal cargo has led to the identification of progressively active agents applicable to cancer immunotherapies. Preclinical studies investigating the role of exosomes in cancer immunotherapy have been extensive,^135^, ^136^ including studies on glioblastoma patients undergoing anti‐survivin immunotherapy. Notably, low levels of specific exosomes have been correlated with sustained patient survival.^137^ Tumour cell‐derived exosomes carrying DNA strands can induce immune cell responses through the STING/cGAS pathway, making them potential players in checkpoint immunotherapy.^138^

DCs‐derived exosomes from cancer patients have shown promise in small clinical trials, providing more precise and cost‐effective immunity against cancer cells compared to other therapies.^139^ In a phase I clinical trial, autologous DCs‐derived exosomes were administered as a vaccination against metastatic melanoma, proving safe. However, further investigation is needed to understand the mechanisms of vaccine antigen distribution and the induction of CD4+ and CD8+ T cell responses.^140^ DCs‐derived exosomes have also demonstrated the ability to increase NK lysis activity and induce specific T‐cell responses in patients with NSCLC,^141^ enhancing anti‐tumour immunity. Combining DCs‐exosome vaccines with NK‐based therapies may yield synergistic immunogenic effects against NK‐dependent tumours.

Immortalized DC lines, engineered to express specific MHC II molecules and/or no MHC proteins, offer the potential for constant and cost‐effective production of DC‐exosomes, reducing therapy costs and culture times. In the context of brain cancer resistant to immune cell recruitment, DCs‐exosomes have shown promise against glioma in mouse models, suggesting their potential as a novel therapy for glioblastoma.^142^ As per current investigational results and clinical trials, exosomes are being explored as potential immunotherapeutic vaccines for various cancers. Notably, autologous DCs‐exosomes have been used in clinical trials meeting current GMP standards, demonstrating their feasibility and short‐term safety. However, safety concerns regarding therapies involving exogenous exosome‐based products may require more rigorous scrutiny. While some investigations have outlined protocols for bulk exosome production and advancements in biocompatibility, additional preclinical and clinical scrutiny is needed to ensure their effectiveness and safety. Importantly, the relatively smaller size and uniform shape of exosomes enable them to evade clearance by the mononuclear phagocyte system, prolonging their circulation time and enhancing their effectiveness in cell‐to‐cell communication. In conclusion, exosomes hold significant promise in developing immunotherapeutic vaccines for diverse diseases, with ongoing clinical trials offering valuable insights into their application and safety considerations.^93^, ^143^, ^144^

### 4.4 Immunomodulation therapy

Exosomes hold significant potential as promising therapeutic agents for treating inflammatory disorders, owing to their favourable attributes such as low immunogenicity, inherent anti‐inflammatory properties and drug delivery capabilities. Notably, using MSC‐derived exosomes in patients with graft versus host disease has shown promising results by reducing the pro‐inflammatory cytokine response (NCT04213248).^145^ Building on this success, ongoing trials are exploring the use of MSC‐derived exosomes for their immunomodulatory properties in the treatment of patients with type I diabetes mellitus (NCT02138331) and macular degeneration (NCT03437759).

Exosomes derived from progenitor cells of specific tissues exhibit superior therapeutic effects for diseases impacting the same tissue, owing to their uniquely specialized molecular composition. These exosomes are rich in bioactive molecules like proteins, lipids and microRNAs that are optimized for the repair and regeneration of the tissue from which they originate, making them highly effective in modulating the local cellular environment.

By facilitating paracrine signalling, these exosomes carry growth factors and cytokines that directly influence neighbouring cells, promoting healing and reducing inflammation. For example, lung progenitor cell exosomes have a superior ability to modulate immune responses, balancing pro‐ and anti‐inflammatory signals, which is particularly vital in lung diseases where inflammation plays a significant role. Cells within the lung also tend to preferentially absorb exosomes from lung progenitor cells due to receptor‐ligand interactions, enhancing the targeted therapeutic effect.

The regenerative properties of these exosomes include promoting cell survival, proliferation and differentiation, which is especially beneficial in conditions like pulmonary fibrosis, where tissue regeneration is compromised. They also reduce fibrosis by inhibiting fibroblast activation and inducing apoptosis in activated myofibroblasts, which are key drivers of the fibrotic process.^146^ MSCs stand out as the most common source of therapeutic exosomes, particularly in regenerative medicine and immunomodulation. For instance, the phase I trial (NCT02138331) led by Nassar et al. investigated the effects of MSC‐derived exosomes from umbilical cord blood on β‐cell mass in type 1 diabetes mellitus. Encouragingly, cord blood‐derived multipotent stem cells have demonstrated successful modulation of the autoimmune response against β‐cells by increasing the number of specific regulatory T cell (Treg) lymphocytes, thus restoring the Th1/Th2 immune balance.^147^, ^148^ Although the results of this trial are yet to be disclosed, the therapeutic potential of exosomes as immunomodulatory agents offers promising prospects in the field of inflammatory disorder treatment.^149^ Some of recent published or ongoing clinical trials related to exosome therapy are mentioned in Table 2.

**TABLE 2:** Recent published or ongoing clinical trials related to exosome therapy.

Therapy	Disease	Source and cargo of exosome	Phase	Clinical outcomes/clinical dose	Number of Clinical Trials	Ref.
Cancer Therapy	Non‐Small Cell Lung Cancer (NSCLC)	Second‐generation dendritic Cell/tumour antigens	II	IFN‐g‐Dex in inoperable NSCLC shows enhanced PFS, 15‐month OS and promising stabilization, indicating potential as maintenance immunotherapy/1.26 × 10^13^–1.67 × 10^14^	NCT01159288	132
Cancer Therapy	Glioma	Autologous Glioma Cell/Anti‐sense molecule targeting ILF1R	I	Current trial was well‐tolerated, indicating favourable median survival, supporting its promise for anti‐tumour immunity.	NCT01550523	132
Cancer Therapy	Pancreatic Cancer (KRASG^12D^ mutation)	Mesenchymal stem cell/Kras^G12D^‐specific siRNA	I	Evaluating safety, efficacy and pharmacokinetics of exosome‐based oncogene inhibition in pancreatic cancer, including determining the maximum tolerated dose and assessing toxicities	NCT03608631	132
Cell‐free therapy	Severe lung diseases, including severe pneumonia caused by P. aeruginosa	Nebulized Adipose‐Derived MSC‐EVs	I	Aerosol inhalation of MSCs‐Exo Enhancing Survival and Tolerability in murine lung injury model induced by Pseudomonas aeruginosa/2 × 10^8 to 16 × 10^8^	NCT04313647	132
Immunomodulation Therapy	Type I Diabetes Mellitus	Umbilical Cord Blood‐derived MSCs	II/III	A possible reduction in the inflammatory state, improvement in β‐cell mass and enhanced glycemic control//(1.22–1.51) × 10^6^ per Kg	NCT02138331	132
Immunomodulation Therapy	Macular Degeneration	Human umbilical Cord Blood‐derived MSCs	I	Results not disclosed: Investigating MSC‐Exos for immunomodulation to promote healing of large and refractory macular holes.	NCT03437759	132
Immunomodulation Therapy	Dry Eye in patients with Graft versus Host Disease	Umbilical MSCs/miR‐204 (engineered exosome containing miR‐124)	I/II	UMSCs ‐exo eye drops improved GVHD‐associated dry eye by reducing inflammation and enhancing recovery, identifying miR‐204 as a key mediator and potential treatment target.	NCT04213248	132
Drug Delivery	Cerebrovascular Disorders	MSCs/miR‐124 (engineered exosome containing miR‐124)	I/II	Improved neural functional recovery, neurogenesis and angiogenesis observed in patients with acute ischemic stroke.	NCT03384433	132
Drug delivery	COVID‐19 infection	Human embryonic kidney T‐REx™‐293 cells engineered to express CD24 (CD24‐Exosomes)/CD24	I	Advancing COVID‐19 Treatment: Positive Clinical Outcomes and Safety Profile of Inhaled EXO‐CD24 in Moderate to Severe Cases, Implications for Phase 3 Investigation	NCT04747574	132
Drug delivery	Early‐Stage Novel Coronavirus Pneumonia	Allogenic COVID‐19 T Cell (CSTC‐Exo)/IFN gamma against COVID‐19 specific fragment peptides	I	Aerosol Inhalation of the Exosomes seems promising to reduce COVID‐19 death risks/2.0 × 10^8^ nano vesicle	NCT04389385	132

Abbreviations: COVID‐19, Coronavirus Disease of 2019; CSTC‐Exos, COVID‐19‐specific T‐cell‐derived Exosomes; Dex, dendritic cell‐derived exosomes; EVs, Extracellular Vesicles; GVHD, Graft versus Host Disease; IFN‐g, Interferon‐gamma; MSCs‐Exo, exosomes derived from mesenchymal stem cells.

### 4.5 Exosomes as therapeutic agents in the drug delivery system: a direct approach for disease treatment

Direct therapeutic approaches harness the potential of exosomes as drug carriers for treating diseases and injuries by exploiting their ability to transfer proteins and nucleic acids between cells. In a clinical trial (NCT03384433),^150^ patients with acute ischemic stroke received treatment with exosomes derived from MSCs. These exosomes were engineered to contain the microRNA miR‐124, known for its role in promoting neurogenesis and ameliorating brain injuries.^151^ The results demonstrated that these modified exosomes improved neural functional recovery as well as promoted neurogenesis and angiogenesis following the stroke.^152^ Throughout a 12‐month follow‐up period, physiological adverse events, including brain edema, seizures and stroke recurrence, were closely monitored.^153^

Exosomes have the potential to act as effective drug carriers capable of penetrating the blood–brain barrier and reaching the central nervous system, owing to their diminutive size.^154^ Notably, experiments with exosomes generated from MSCs have shown promising results in promoting neurogenesis and facilitating cognitive function recovery in mice with Alzheimer's (AD). Furthermore, the therapeutic use of exosomes derived from human umbilical cord MSCs has been approved to mitigate neuroinflammation and enhance therapeutic efficacy in AD‐affected mice by repairing cognitive dysfunctions and reducing amyloid beta protein (Aβ) deposition.^155^, ^156^ Based on the promising results observed in these clinical trials, several biotechnology companies, including Celltex Therapeutics, are actively advancing the development of MSC‐derived exosomes (MSC‐Exo)‐based treatments specifically targeted at addressing Alzheimer's disease. Additionally, Aruna Bio is exploring the use of neural stem cell‐derived exosomes to overcome the blood–brain barrier, while Codiak BioSciences is at the forefront of pioneering an innovative exosome‐based vaccine platform to prevent infectious diseases. These advancements in exosome‐based treatments hold significant potential for addressing a wide range of neurological disorders and infections.^6^

## 5 CONCLUSION

Exosome release is essential for medicinal drugs that target gene transfer and specific cell types. The state of the cells from which exosomes originate influences their release, composition and content. Despite the emergence of novel technologies, existing exosome isolation and characterization techniques are widely employed for prognostic and diagnostic applications. This article outlines the biological functions, isolation, characterization and potential therapeutic applications of exosomes in drug delivery. The biogenesis of exosomes involves various cellular machinery, including different proteins and lipids, which vary depending on the cell type and cellular homeostasis.

Additionally, this article covers the fundamental principles of several exosome isolation and characterization techniques. Exosome properties, such as size and number, can be analysed using DLS and NTA approaches. While electron microscopy is the most effective method for examining the structural qualities of exosomes, an ideal approach would allow simultaneous examination of both the biological and structural aspects using a single instrument. Therefore, the purification and characterization of exosomes require more advanced techniques. To enhance the quality of exosome isolation and ensure the reliability of their applications, appropriate techniques for exosome collection and characterization must be employed. One of the primary technological challenges in exosome detection for therapeutic purposes is distinguishing exosomes derived from normal cells from those isolated from damaged cells. Furthermore, the inherent heterogeneity of exosomes poses an additional challenge. Developing a combination of different quantification methods may be necessary to distinguish between exosome subtypes in heterogeneous samples.

Therefore, exosome detection and quantification hold great promise for new opportunities. A crucial aspect of new techniques should be the ability to separate distinct vesicle subpopulations, allowing for assigning specific functions and origins to each subpopulation. Developing novel techniques to regulate exosome biogenesis, isolation, composition, secretion and interaction will significantly contribute to our understanding of their function. Moreover, overcoming these limitations will pave the way for creating innovative treatment approaches that leverage the therapeutic potential of exosomes.

## AUTHOR CONTRIBUTIONS

**Farnaz Sani:** Conceptualization (equal); investigation (equal); validation (equal); writing – original draft (equal); writing – review and editing (equal). **Faezeh Shafiei:** Data curation (equal); investigation (equal); visualization (equal); writing – original draft (equal). **Farshad Dehghani:** Data curation (equal); investigation (equal); writing – original draft (equal). **Yasaman Mohammadi:** Data curation (equal); investigation (equal); methodology (equal); validation (equal). **Mohammadhossein Khorraminejad‐Shirazi:** Investigation (equal); methodology (equal); visualization (equal). **Abbas Fazel Anvari‐Yazdi:** Data curation (equal); investigation (equal); validation (equal). **Zahra Moayedfard:** Methodology (equal); resources (equal). **Negar Azarpira:** Conceptualization (equal); funding acquisition (equal); project administration (equal); validation (equal). **Mahsa Sani:** Conceptualization (equal); investigation (equal); project administration (equal); validation (equal); visualization (equal).

## FUNDING INFORMATION

None.

## CONFLICT OF INTEREST STATEMENT

The authors declare that they have no competing interests.

## CONSENT FOR PUBLICATION

Not applicable.

## Data Availability

All data is available in this article.
